# Atypical Sensory Processing Is Associated With Lower Body Mass Index and Increased Eating Disturbance in Individuals With Anorexia Nervosa

**DOI:** 10.3389/fpsyt.2022.850594

**Published:** 2022-03-31

**Authors:** Emma Saure, Tuulia Lepistö-Paisley, Anu Raevuori, Marja Laasonen

**Affiliations:** ^1^Department of Psychology and Logopedics, Faculty of Medicine, University of Helsinki, Helsinki, Finland; ^2^Child Neurology, Helsinki University Hospital, University of Helsinki, Helsinki, Finland; ^3^Clinicum, Department of Public Health, University of Helsinki, Helsinki, Finland; ^4^Department of Adolescent Psychiatry, Helsinki University Hospital, Helsinki, Finland; ^5^Logopedics, Philosophical Faculty, School of Humanities, University of Eastern Finland, Joensuu, Finland

**Keywords:** anorexia nervosa, feeding and eating disorders, autism spectrum disorder, sensory processing, selective eating

## Abstract

**Background:**

Elevated autism spectrum disorder (ASD) traits are associated with anorexia nervosa (AN). Conversely, eating disturbances, which are core characteristics of AN, are common in ASD. Among individuals with ASD, atypical sensory processing is associated with eating disturbance. Because AN and ASD appear to overlap, it would be crucial to understand whether sensory processing atypicality exist also in AN. Further, it would be essential to find if atypical sensory processing is associated with eating disturbances in individuals with AN, since treatment modifications may be needed. We therefore aimed to examine whether atypical sensory processing is associated with AN and its core characteristics.

**Methods:**

Participants of the current study included 42 individuals with AN and 40 healthy controls (HCs). All participants were adult women. Sensory processing, other ASD traits, and eating disorder symptoms were assessed with self-report questionnaires.

**Results:**

Individuals with AN reported lower registration, decreased sensation seeking, increased sensory sensitivity, and increased sensation avoiding compared to HCs. When analyzing groups with restrictive AN (AN-R) and binge-purge type AN (AN-BP) separately, only individuals with AN-R exhibited decreased sensation seeking, and only those with AN-BP exhibited lower registration. After controlling for body mass index as a covariate, group differences remained significant only in sensory sensitivity between individuals with AN and HCs. Increased atypical sensory processing predicted lower body mass index and increased disordered eating.

**Conclusion:**

Results suggest that sensory processing appears to differ between AN and HC women, and AN subtypes may exhibit distinct sensory processing atypicality. Sensory sensitivity may be stable traits whereas other aspects of atypical sensory processing may be related to acute AN. Atypical sensory processing may contribute to the severity of AN, and thus it is crucial to recognize sensory processing differences when treating individuals with AN.

## Introduction

Anorexia nervosa (AN) is a potentially severe mental health disorder which core characteristics are restriction of energy intake leading to low body weight, intense fear of gaining weight, or persistent behavior that interferes with weight gain and a disturbance in the way in which one's body weight/shape is experienced (1). Elevated autism spectrum disorder (ASD) traits and diagnosed ASD are overrepresented among individuals with AN (2–5). ASD is a developmental condition characterized by deficits in social interaction and communication, as well as repetitive, stereotyped behavior and interests (1). Atypical sensory processing, for instance, atypical responses to touch, pain, smell, taste, or bodily-related perception, is a common feature and a diagnostic criterion of ASD (1). In individuals with ASD, atypical sensory processing is strongly associated with eating disturbances, which most commonly include selective eating, food aversion due to specific texture or color, and food neophobia (6–9). Because disordered eating is a core feature in AN, and ASD and AN appear to somewhat overlap in their disordered eating symptoms, it would be essential to understand whether disordered eating in AN is also associated with the level of atypical sensory processing. However, only a few previous studies have investigated atypical sensory processing among individuals with AN, and the possible joint effect of high ASD traits for different sensory processing dimensions has not been taken into account (10–12).

Sensory processing refers to the ability to register and modulate sensory information and to organize and use this information to respond to environmental stimuli. It consists of two constructs: neurological threshold to sensory stimuli and response strategy to these stimuli (13). Response strategies for sensory stimuli refer to ways of regulating perceived sensory input. For example, individual may stay away from noisy places, leave to another place when smelling a strong odor, eat only bland food or avoid some food textures in order to minimize sensory input. Two constructs combine into four basic dimensions of sensory processing: low registration, which is characterized by a low threshold and passive regulation; sensation seeking that is characterized by a high threshold and active regulation; sensation sensitivity that is characterized by a low threshold and passive regulation, and sensation avoiding that is characterized by a low threshold and active regulation.

Some studies have shown that individuals with AN with high ASD traits self-report increased sensory sensitivity and sensation avoiding (11, 12, 14), whereas one study did not found association between ASD traits and taste or smell processing in individuals with AN (15). The two subtypes of AN may differ from each other in ASD traits and exhibit a different pattern of sensory processing. Restrictive anorexia nervosa (AN-R) symptoms include food restriction without binge-eating and/or purging behavior; and binge-purge type anorexia nervosa (AN-BP) symptoms include both food restriction and binge-eating and/or purging (1). Individuals with AN-R have been reported to exhibit higher self-reported ASD traits and more ASD-related neuropsychological characteristics than individuals with AN-BP (16, 17). In addition, a recent study found that only individuals with the AN-R subtype reported more hyper-hyporeactivity to sensory input than HCs, whereas those with AN-BP were comparable to HCs (16). Unfortunately, the study did not investigate specific dimensions of sensory processing or specify if AN-R participants exhibited more hyper- or hyporeactivity than HCs; instead these were combined under the same scale. In summary, it is likely that specifically individuals with AN-R have elevated ASD traits and difficulties in sensory processing. However, we are not aware of any previous studies delineating the specific dimensions of sensory processing difficulties by the two subtypes of AN or characteristics of AN.

Sensory processing may be associated with the core symptoms of AN, that is, food restriction and body image distortion, that are shared with both its subtypes. One study found that atypical sensory processing among individuals with AN positively correlated with the severity of overall eating disorder symptoms (10). However, the possible joint effect of other ASD traits and atypical sensory processing was not investigated nor the role of the subtypes of AN. In another recent study including participants with both AN and ASD, the participants described that they had food-specific sensory sensitivities, and that food restriction was often related to the sensory properties of food (18). However, since this study was based on qualitative interviews, it did not investigate the possible association of sensory sensitivity and eating disorder symptoms with quantitative methods. In addition to eating problems, sensory processing may be involved in another core symptom of AN, that is, body image disturbances. Body image (i.e., subjective representation of one's own body) is partly based on sensations arising within the body, for example, sensing body position and body boundaries (19). To sum up, atypical sensory processing may have a central role in understanding the symptoms of AN.

In conclusion, subtypes of AN may exhibit different patterns of sensory processing, but studies investigating this are lacking. Further, sensory processing difficulties may relate to the core eating disorder symptoms, but the evidence is scarce. High ASD traits in AN are associated with illness prolongation and poorer treatment outcomes (20–23). Treatment adaptations could therefore be beneficial for individuals with AN with high ASD traits. Further, if the AN subtypes exhibit different sensory processing patterns, treatment modifications according to subtypes may also be necessary. The present study aimed to examine: (1) whether sensory processing dimensions differ among individuals with AN and healthy controls (HCs); and further, whether there are differences in sensory processing between individuals with two subtypes of AN (AN-R and AN-BP), and (2) whether atypical sensory processing predicts core characteristics of AN [eating disorder symptoms, body mass index (BMI)] or duration of illness when the group (AN or HC) and other ASD traits are also included in the analysis.

## Materials and Methods

### Participants

Participants with AN were recruited via The Eating Disorder Association of Finland and via Turku University Hospital. All individuals with AN had been diagnosed by professionals and fulfilled the diagnostic criteria of AN or atypical AN (F50.0 and F50.1 in ICD-10 World Health Organization), (24), both of which are included in DSM-5 AN [307.1, (1)]. Participants with AN were asked to report in the background questionnaire whether their symptoms included binge and/or purge behavior. Those who reported binge and/or purge behavior were included AN-BP group, and those who did not report those behaviors were included AN-R group. These subtypes are not defined in ICD-10, which is used in Finland, and therefore the subtypes were not defined by professionals. HCs were recruited via the University of Helsinki emailing lists. The participants participated voluntarily after providing informed written consent for the study. The Ethics committee of Helsinki Uusimaa Hospital District (HUS/1886/2017) approved the study and the study was conducted in accordance with the Declaration of Helsinki.

Exclusion criteria for all the participants were as follows: history of diagnosed psychosis or substance abuse, ASD, ADHD, neurological disorder, learning disability, head trauma with unconsciousness, or score of under 70 in full-scale intelligence quotient (FSIQ), verbal comprehension index (VCI), or perceptual reasoning index [PRI, (25)]. Additionally, for the HC participants, exclusion criteria included any current or past diagnosed mental disorder. All participants were adult females. The final sample comprised 82 participants: 40 HCs and 42 individuals with AN, of whom 20 had AN-R and 22 AN-BP. Three individuals were excluded from the original HC group (*n* = 43): one had a PRI score under 70, one reported having a diagnosed mental disorder, and one reported having a diagnosed neurological condition. The characteristics of participants are presented in Table 1.

**Table 1:** Characteristics of the AN and HC groups.

	**AN (*n* = 42)**	**AN-R only (*n* = 20)**	**AN-BP only (*n* = 22)**	**HC (*n* = 40)**
	**mean (SD)**	**mean (SD)**	**mean (SD)**	**mean (SD)**
Age (years)	23.61 (3.69)	24.34 (3.59)	22.95 (3.74)	23.10 (3.03)
Education (years)	14.21 (2.43)	14.55 (2.56)	13.91 (2.31)	14.15 (1.82)
Full-scale intelligence quotient (FSIQ, WASI)	108.31 (17.15)	110.95 (17.39)	105.91 (16.97)	109.68 (13.00)
Perceptual reasoning index (PRI, WASI)	105.33 (15.91)	107.65 (16.71)	103.23 (15.22)	106.10 (15.02)
Verbal comprehension index (VCI, WASI)	108.36 (14.37)	110.00 (14.70)	106.86 (14.25)	110.48 (13.91)
Autism spectrum traits (AQ)	21.57^***^ (9.29)	22.05^***^ (9.10)	21.14^***^ (9.65)	10.90 (6.32)
Body mass index	17.00^***^ (2.64)	16.68^***^ (2.88)	17.30^***^ (2.44)	21.61 (1.22)
Duration of illness	7.46 (3.65)	8.14 (3.83)	6.85 (3.45)	N/A
Eating disorder symptoms (EDE-Q)	85.86^***^ (33.08)	78.90^***^ (36.12)	92.18^***^ (29.46)	9.32 (7.11)
Eating disturbances associated with ASD (SWEAA)	91.38^***^ (35.51)	101.50^***^ (26.47)	82.18^***^ (27.03)	35.51 (11.75)
Psychopharmacological medication *n* (%)	16^***^ (38.1 %)	7^***^ (35.0)	9^***^ (40.9%)	0 (0 %)
Comorbid psychiatric conditions *n* (%)	25^***^ (59.5 %)	11 ^***^ (55%)	14^***^ (63.6%)	0 (0 %)

*Means and standard deviations are presented in the table. Significant between-group differences when compared to HCs are marked with the following symbols:*

*** *p < 0.001*.

*Participants with AN-R and AN-BP did not differ from each other in any of the variables*.

*WASI, Wechsler Abbreviated Scale of Intelligence; FSIQ, Full-Scale Intelligent Quotient; VCI, Verbal Comprehensive Index; PRI, Perceptual Reasoning Index; AQ, Autism Quotien; EDE-Q, Eating Disorder Examination Questionnaire; SWEAA, Swedish Eating Assessment for Autism spectrum disorders*.

### Measures

#### Background Information

Participants filled in a background questionnaire, in which they were asked about their date of birth, education in years, existing psychiatric and neurological conditions, psychopharmacological medication, weight, and height. BMI was calculated using self-reported weight and height.

#### Adult Sensory Profile

The Sensory Profile is a widely used self-report questionnaire that measures sensory processing (25). It consists of 60 items that are self-rated for the frequency of a range of behaviors on a five-point scale. The scale has four subscales: low registration (questions about situations in which individuals do not notice some sensory stimulus, e.g., do not notice when someone touches them), sensory seeking (questions about behavior that lead to sensory stimulation, e.g., if individual likes to go places that have bright lights and loud music or if individual likes eat spicy food), sensory sensitivity (questions about how easily individual is distracted if there is a lot of sensory stimulation, e.g., loud noises or particular food textures) and sensory avoiding (questions about behavior that lead muting of sensory stimulation, e.g., using earplugs, staying away from noisy places, eating only familiar food). Higher scores indicate lower registration, sensory seeking, sensory sensitivity, and sensory avoiding.

#### Wechsler Abbreviated Scale of Intelligence

WASI was used to assess participants' cognitive ability. WASI consists of the following four subtests: vocabulary, similarities, block design, and matrix reasoning. The WASI produces verbal, performance, and full-scale intelligence quotient scores (VCI, PRI, FSIQ) (26). There is no official edition of the WASI in Finland, so these quotients were formed based on the same subtests from the Wechsler Adult Intelligence Scale-IV (WAIS-IV, Wechsler), (27).

#### Eating Disorder Examination Questionnaire

Eating Disorder Symptoms were assessed with EDE-Q (28, 29). EDE-Q is a self-report questionnaire consisting of four subscales measuring restraint, eating concerns, shape concerns, and weight concerns. Higher scores indicate more eating disorder symptoms.

#### Swedish Eating Assessment for Autism Spectrum Disorder

SWEAA is a self-report questionnaire designed to measure eating disturbances associated with ASD (30). SWEAA measures eating problems related to perception, motor control, purchase of food, eating behavior, mealtime surroundings, social situations at mealtime, and recognition of hunger and satiety. Higher scores indicate more eating problems.

#### Autism Quotient

ASD traits were measured using AQ that is a widely used scale containing 50 self-report items (31, 32). It measures the following ASD traits: social skills, attention switching, attention to details, communication, and imagination. Higher scores indicate increased ASD traits. AQ was used in the analysis to control for other ASD traits beyond atypical sensory processing.

### Data Analysis

Power analysis revealed that the sample size was sufficient for sensation seeking, sensation sensitivity, and sensation avoiding (80% power with alpha set at *p* < 0.05) (33). In the context of low registration, our study was slightly unpowered as about 60 participants were required. The normality of variables in the two samples was assessed with histograms and with Shapiro-Wilk tests. ANOVA was used to calculate differences for those background variables that were normally distributed in all groups (FSIQ, VCI, illness duration, BMI). Kruskal-Wallis tests were used for calculating group differences in the other background variables (age, education, PRI, EDE-Q, SWEAA, AQ). For dichotomous variables, that is, psychopharmacological medications and comorbid conditions, group differences were calculated with the chi-square test.

Subscales of Sensory Profile were normally distributed in all three groups. All subscales were first analyzed together with multivariate analysis of variance (MANOVA), and secondly, separately with univariate analysis of variance (ANOVA). The scores of the sensory profile subscales were included in the analyses as dependent variables. The group (in the first analyses AN and HC groups, and in the second analyses AN-R, AN-BP, and HC groups) was included in analyses as an independent variable. Bonferroni correction was used for multiple comparisons. BMI was included as a covariate since it differed significantly between the groups.

Linear regression analyses were used to investigate whether atypical sensory processing (composite score) predicts eating disorder characteristics (BMI, duration of illness, scores of EDE-Q, scores of SWEAA). The group (AN or HC) was entered at the first step, and in addition, ASD traits (AQ) were added to the model at the second step, in order to control for the more general prediction by them. Sensory processing (composite score) was entered at the third step as an independent variable. Dependent variables in separate analyses were BMI, duration of illness scores of SWEAA, and scores of EDE-Q. Before the regression analyses, scores of sensory processing dimensions (low registration, sensation seeking, sensory sensitivity, and sensation avoiding) were summed up and combined under one composite score representing overall atypical sensory processing. Results of sensation seeking were reversed (scores were subtracted from 75 that is the maximum score of the scales) because in the other dimensions, higher scores indicate higher atypicality, whereas, in the sensation seeking scale, lower scores indicate higher atypicality. The internal consistency of the sensory processing composite was adequate when investigated with an inter-item correlation matrix and Cronbach's Alpha of 0.789.

In the SWEAA, the data of five HC participants were missing because the questionnaire was included after these participants had already participated in the study. Weight information was missing from two participants' self-report questionnaires (one AN-R and one AN-BP). Missing information was not replaced, and thus these participants were not included in the analysis concerning the missing variables (SWEAA or BMI). The Statistical Package for the Social Sciences, version 26.0 (34), was used to analyze the data.

## Results

### Group Differences in Sensory Processing

In the first analysis, we examined with MANOVA whether there were differences between the total AN and HC groups. There was a significant difference between the AN and HC groups in sensory processing [F (4.77) = 17.812, *p* < 0.001, Wilks' λ = 0.519, partial η^2^ = 0.481]. Follow-up ANOVAs revealed significant group differences in all sensory processing variables: when compared to HCs, the AN group exhibited more low registration [F (1.80) = 4.005, *p* = 0.049, partial η^2^ = 0.048], less sensation seeking [F (1.80) = 9.958, *p* = 0.002, partial η^2^ = 0.111], more sensory sensitivity [F (1.80) = 70.216, *p* < 0.001, partial η^2^ = 0.467], and more sensation avoiding [F (1.80) = 39.555 *p* < 0.001, partial η^2^ = 0.331] (see Table 2). After controlling for BMI as a covariate in MANCOVA analysis, the significant group difference remained [F (4.74) = 5.785, *p* < 0.001, partial η^2^ = 0.238]. Follow-up ANCOVAs revealed significant group difference only in sensation sensitivity [F (1.77) = 16.168, *p* < 0.001, partial η^2^ = 0.174], whereas in other sensory processing dimensions, AN and HC groups did not differ significantly from each other. See Supplementary Tables 1, 2 for summarized results of MANOVA, MANCOVA, ANOVAs and ANCOVAs.

**Table 2:** Means and standard deviations of sensory processing dimensions are presented in the table.

	**AN (*n* = 42)**	**AN-R only (*n* = 20)**	**AN-BP only (*n* = 22)**	**HC (*n* = 40)**
	**mean (SD)**	**mean (SD)**	**mean (SD)**	**mean (SD)**
Low registration	31.76^*^ (7.75)	29.30 (7.82)	34.00^*^ (7.13)	28.65 (6.20)
Sensation seeking	42.60^**^ (8.38)	41.05^**^ (6.82)	44.00 (9.52)	47.90 (6.71)
Sensation sensitivity	48.62^***^ (7.84)	47.90^***^ (8.22)	49.27^***^ (7.62)	35.63 (6.03)
Sensation avoiding	44.19^***^ (10.04)	45.55^***^ (9.23)	42.95^***^ (10.79)	32.90 (5.43)

*Statistical significance in ANOVA post-hoc analyses when comparing the AN-R and AN-BP groups to HCs is marked with the following symbols:*

* *p < 0.05*

** *p < 0.01*

*** *p < 0.001*.

In the second analysis, we examined with MANOVA whether there were differences between AN-R, AN-BP, and HC groups. There was a significant difference between the AN-R, AN-BP, and HC groups in sensory processing [F (8.152) = 9.333*, p* < 0.001, Wilks' λ = 0.450 partial η^2^ = 0.329]. Follow-up ANOVAs revealed significant group differences in all sensory processing variables, that is, low registration [F (2.79) = 4.550, *p* = 0.013, partial η^2^ = 0.103], sensation seeking [F (2.79) = 5.809, *p* = 0.004, partial η^2^ = 0.128], sensory sensitivity [F (2.79) = 35.043, *p* < 0.001, partial η^2^ = 0.470], and sensation avoiding [F (2.79) = 20.330, *p* < 0.001, partial η^2^ = 0.340]. In Bonferroni corrected *post-hoc* analyses, the AN-R group did not differ significantly from HCs in low registration but exhibited significantly less sensation seeking (*p* = 0.004) as well as significantly more sensation sensitivity (*p* < 0.001) and more sensation avoiding (*p* < 0.001) than HCs. The AN-BP group did not differ significantly from HCs in sensation seeking but exhibited significantly more low registration (*p* = 0.013), more sensation sensitivity (*p* < 0.001) and more sensation avoiding (*p* < 0.001) than HCs (see Table 2). The AN-R and AN-BP groups did not differ significantly from each other in any of the investigated sensory processing dimensions. After controlling for BMI as a covariate in MANCOVA analysis, the significant group difference remained [F (8.146) = 4.451, *p* < 0.001, partial η^2^ = 0.196]. Follow-up ANCOVAs revealed significant group difference only in sensation sensitivity [F (2.76) = 8.641, *p* < 0.001, partial η^2^ = 0.185] whereas AN-R, AN-BP and HC groups did not differ significantly from each other in other sensory processing dimensions. Further, pairwise comparisons revealed that both AN-R and AN-BP groups exhibited significantly more sensory sensitivity than HCs (*p* = 0.004 and *p* < 0.001, respectively). See Supplementary Tables 1, 2 for summarized results of MANOVA, MANCOVA, ANOVAs and ANCOVAs.

### Sensory Processing Atypicality as a Predictor for Eating Disorder Characteristics

Linear regression revealed that group at step 1 was a significant predictor for BMI [F (1.78) = 100.250, *p* < 0.001] and ASD traits at step 2 enhanced significancy of the prediction [F (2.77) = 56.525, *p* < 0.001; F change (1.77) = 6.164, p change = 0.015]. At step 3, when sensory processing atypicality was included as a third predictor, the accuracy of prediction was further significantly increased [F (3.76) = 41.314*, p* < 0.001; F change (1.76) = 5.007, p change = 0.028], indicating that higher sensory processing atypicality was associated with lower BMI after the prediction by group and other ASD traits were also included in the model. Only the group (AN or HC) predicted significantly the duration of illness [F (1.80) =166.921, *p* < 0.001], and adding ASD traits or sensory processing atypicality to the model did not significantly increase the accuracy of prediction [F (2.79) = 84.053, *p* < 0.001; F change (1.79) = 1.060, p change = 0.306; F (3.78) = 58.526, *p* < 0.001; F change (1.78) = 3.070, p change = 0.084., respectively]. Also, for the eating disorder symptoms measured by EDE-Q, only the group (AN or HC) was a significant predictor [F (1.80) =205.016, *p* < 0.001] while ASD traits and sensory processing atypicality did not significantly increase the accuracy of prediction [F (2.79) = 105.189, *p* < 0.001; F change (1.79) = 2.224, p change = 0.140; F (3.78) = 69.891, *p* < 0.001; F change (1.78) = 0.535, p change = 0.467, respectively]. For the eating disorder symptoms measured by SWEAA, the group at step 1 [F (1.75) = 119.941, *p* < 0.001], and ASD traits at step 2 enhanced significancy the prediction [F (2.74) = 83.698, *p* < 0.001; F change (1.74) = 18.873, p change < 0.001], and when sensory processing atypicality were included at step 3 the accuracy of interpretation was further significantly increased [F (3.73) = 65.178, *p* < 0.001; F change (1.73) = 9.319, p change = 0.010], indicating that higher sensory processing atypicality was associated with severity of eating disorder symptoms measured by SWEAA after the prediction by group and other ASD traits were also included in the model (see Table 3 for total R^2^ for every step and R^2^ changes).

**Table 3:** Coefficient of determination (R^2^) for each step and changes in R^2^ are presented in table.

	**Predictors**	**BMI**	**Duration of illness**	**EDE-Q**	**SWEAA**
First step: group	R^2^ (only group)	0.562^***^	0.672^***^	0.719^***^	0.615^***^
Second step: group and ASD traits	R^2^ change	0.032^*^	0.004	0.008	0.078^***^
	R^2^ (group and ASD traits)	0.595^***^	0.672^***^	0.727^***^	0.693^***^
Third step: group, ASD traits, and sensory processing	R^2^ change	0.025^*^	0.012	0.002	0.035^**^
	R^2^ (group, ASD traits and sensory processing)	0.620^***^	0.681^***^	0.729^***^	0.728^***^

*Statistical significance is marked with following symbols:*

* *p < 0.05*

** *p < 0.01*

*** *p < 0.001*.

## Discussion

This study aimed to investigate sensory processing among individuals with AN. We found that women with AN had significantly lower registration, less sensation seeking, and increased sensory sensitivity as well as sensation avoiding compared to healthy control individuals. Examination of the two subtypes of AN showed that only the participants with AN-R exhibited significantly less sensation seeking, and only the participants with AN-BP exhibited significantly lower registration, as compared to HCs. However, after controlling for BMI, significant group differences remained only in sensory sensitivity between individuals with AN and HCs. Both individuals with AN-R and AN-BP exhibited heightened sensory sensitivity when compared to HCs. Higher ASD traits and atypical sensory processing predicted lower BMI and higher eating disturbance after the group was also included as a predictor.

In line with the previous studies (11, 12), we found that individuals with AN had high sensation sensitivity. These difficulties could underlie decreased eating among individuals with AN, since eating often includes plenty of sensory stimuli, including the texture, smell, taste, and temperature of food, sounds of eating, and visceral sensations caused by food (e.g., stomach fullness). Individuals with sensory sensitivities may perceive eating as unpleasant because of its sensory load, and in order to avoid unpleasant sensory experiences, they may refrain from eating (35). Further, we found that AN-R and AN-BP differed in sensory processing when compared to HCs. Both subgroups exhibited more sensation sensitivity and sensation avoiding than HCs, whereas only the AN-R subgroup exhibited less sensation seeking than HCs. Low sensation seeking among AN-R could reflect the same phenomena discussed above that sensory input is perceived as unpleasant. Binge-purge episodes seen in AN-BP are accompanied by sensory stimulation and can be seen as a way to seek sensory experiences. Therefore, it is possible that low sensation seeking acts as a protective factor from these episodes for those with AN-R, whereas those with AN-BP tend to seek sensations. Additionally, only the AN-BP group exhibited lower registration, implying a lower threshold for noticing sensory stimulation, as compared to HCs. Low registration is associated with hyporesponsiveness to sensory stimulation among individuals with ASD (36). Therefore, low registration could also contribute to binge-purge episodes among AN-BP individuals as these episodes include high sensory stimulation.

After controlling for BMI, significant group differences remained only in sensory sensitivity between individuals with AN and HCs. This may indicate that some aspects of atypical sensory processing relate to acute AN. In the context of other ASD traits, many studies have reported that these traits both precede the onset of AN and remain after recovery from it (37–39). Some studies have found elevated ASD traits only during acute AN (40). It is suggested that starvation or other issues related to acute AN may strengthen ASD traits, which may also be true in the context of sensory processing (22). It has been suggested as well that low BMI may heighten the vigilance for sensations (12) that may be one acute AN related factor contributing to sensory processing atypicality. However, it has also been reported that sensory sensitivity and differences in sensory responses persist after weight recovery (10–12, 41). Therefore, sensory sensitivity seems to be a trait, whereas other dimensions of sensory processing may reflect some acute illness related processes.

In this study, we found that more atypical sensory processing predicted eating disturbances after group (AN or HC) and ASD traits were already considered, implying that sensory processing independently contributes to eating disturbances. Interestingly, we found this association only when eating disturbances were measured with SWEAA that includes questions of disordered eating typical to individuals with ASD, such as disturbed eating related to sensory issues, social situations, motor control, selective eating, as well as understanding hunger and satiety signals (30). In contrast, atypical sensory processing did not predict symptoms of eating disorders when measured with the widely used EDE-Q questionnaire, which includes questions about eating, weight, and body shape concerns. We suggest that this indicates that among individuals who exhibit sensory processing difficulties, eating disorder symptoms are qualitatively different from what has traditionally been regarded as eating disorder symptoms in AN, and which are measured by EDE-Q (such as weight and shape concerns). This can be a reason why these individuals have challenges in benefiting from traditional treatment methods (20, 21).

We also found that increased atypical sensory processing predicted lower BMI, indicating increased severity of AN. This is in line with a previous study showing that sensation sensitivity was positively correlated with lower minimum BMI during the eating disorder among those with AN (12). It is, therefore, possible that both high sensory sensitivity and low sensation seeking contribute to low weight. It has also been suggested that a low BMI may mute aversive sensory stimuli and therefore help cope with sensory overload (12). To summarize, sensory processing difficulties may contribute to the core symptoms of AN, including eating disturbance and low body weight, thus contributing to the illness severity.

Atypical sensory processing is likely to manifest specifically among those individuals with AN who have also high ASD traits (14). Our findings suggest that elevated traits of ASD, and particularly atypical sensory processing, predict the core symptoms of AN. Characteristics of ASD in individuals with AN manifesting either as a comorbid ASD diagnosis or increased ASD traits have been found to increase the risk for illness prolongation and poor prognosis (20, 21, 37). Sensory processing difficulties may be one ASD-related factor that contributes to poor prognosis in AN. Therefore, those with high ASD traits could benefit from treatment modifications that take aspects of sensory processing into account.

### Limitations

ASD traits were measured with a self-report questionnaire that is dependent on the participants' ability to recognize and verbalize these traits. Additionally, weight and height were self-reported, and some reporting bias may occur. In addition, the subtyping of AN (AN-R or AN-BP) was based on self-reported symptoms, not on clinical interview or diagnoses made by professionals. Further, comorbid conditions were assessed with self-report questionnaire rather than clinical interview. It is also possible that some comorbid symptoms, especially anxiety and depressive symptoms that are very common in individuals with AN and play an important role in AN symptoms, (42) may also explain some of the group differences in sensory processing. All the participants were women; it is known that both quantitative and qualitative sex/gender differences exist in ASD traits, e.g., females with ASD have less repetitive and restricted behavior than males with ASD, and therefore, males with AN and ASD traits may also exhibit a different pattern of sensory processing (43). Therefore, our results may not be generalizable to men with AN.

## Conclusion

In this study, we found sensory processing differences in individuals with AN compared to healthy individuals. Participants with both subtypes of AN exhibited increased sensory sensitivity and sensory avoiding, whereas only those with AN-R subtype exhibited decreased sensory seeking, and only those with AN-BP subtype exhibited lower registration, as compared to HCs. After controlling for BMI, group differences remained only in sensory sensitivity between individuals with AN and HCs. This may indicate that sensory sensitivity is a stable trait, whereas other aspects of atypical sensory processing may be related to acute AN. Higher ASD traits and increased atypical sensory processing predicted higher ASD-related eating disturbance and lower BMI, implying that sensory processing differences may contribute to illness severity. In the future, it is crucial to recognize atypical sensory processing patterns when treating individuals with AN, especially among those with high ASD traits, and develop treatment modifications for those with AN and atypical sensory processing.

## Data Availability Statement

The datasets presented in this article are not readily available because the data that support the findings of this study are available on request from the corresponding author. The data are not publicly available due to privacy. Requests to access the datasets should be directed to emma.saure@helsinki.fi.

## Ethics Statement

The studies involving human participants were reviewed and approved by the Ethics Committee of Helsinki Uusimaa Hospital District. The patients/participants provided their written informed consent to participate in this study.

## Author Contributions

ES contributed to the conceptualization, conducting the research, data analysis, and writing the original manuscript. TL-P, AR, and ML contributed to the conceptualization and manuscript's editing and revision. All authors contributed to the article and approved the submitted version.

## Funding

This work was supported by the Finnish Cultural Foundation and the Päivikki and Sakari Sohlberg Foundation.

## Conflict of Interest

The authors declare that the research was conducted in the absence of any commercial or financial relationships that could be construed as a potential conflict of interest.

## Publisher's Note

All claims expressed in this article are solely those of the authors and do not necessarily represent those of their affiliated organizations, or those of the publisher, the editors and the reviewers. Any product that may be evaluated in this article, or claim that may be made by its manufacturer, is not guaranteed or endorsed by the publisher.
