# Supplementary material for: Atypical Sensory Processing Is Associated With Lower Body Mass Index and Increased Eating Disturbance in Individuals With Anorexia Nervosa
Source: Front Psychiatry. 2022 Mar 31;13:850594. doi: 10.3389/fpsyt.2022.850594 (PMC9008215; doi:10.3389/fpsyt.2022.850594)
Supplement: Supplementary file 1 [file Data_Sheet_1.pdf]

Supplementary Table 1. Summary of results of MANOVAs and MANCOVAs in sensory processing. Statistical significance is marked with following symbols: * p < 0.05 ** p < 0.01 *** p < 0.001

	**AN vs HC**	**AN vs HC**<br>**BMI as covariate**	**AN-R vs AN-BP vs HC**	**AN-R vs AN-BP vs HC**<br>**BMI as covariate**
F	17.812	5.785	9.333	4.451
p	***	***	***	***
Wilks´ λ	0.519	0.762	0.450	0.646
partial η^2	0.481	0.238	0.329	0.196

Supplementary Table 2. Summary of results of ANOVAs and ANCOVAs in sensory processing dimensions. Statistical significance is marked with following symbols: * p < 0.05 ** p < 0.01 *** p < 0.001

		**AN versus HC**	**AN vs HC**<br>**BMI as covariate**	**AN-R vs AN-BP vs HC**	**AN-R vs AN-BP vs HC**<br>**BMI as covariate**
Low registration	*F*	4.005	0.736	4.550	3.029
	p	*	n.s.	*	n.s.
	partial η^2	0.048	0.009	0.103	0.074
Sensation seeking	*F*	9.958	0.247	5.809	0.656
	p	**	n.s.	**	n.s.
	partial η^2	0.111	0.003	0.128	0.017
Sensation sensitivity	*F*	70.216	16.168	35.043	8.641
	P	***	***	***	***
	partial η^2	0.467	0.174	0.470	0.158
Sensation avoiding	*F*	39.555	3.471	20.330	2.123
	p	***	n.s.	***	n.s.
	partial η^2	0.331	0.043	0.340	0.053
